# Supplementary material for: Distinct brain morphometry patterns revealed by deep learning improve prediction of post-stroke aphasia severity
Source: Commun Med (Lond). 2024 Jun 12;4:115. doi: 10.1038/s43856-024-00541-8 (PMC11169346; doi:10.1038/s43856-024-00541-8)
Supplement: Supplementary file 2 — Supplementary Information [file 43856_2024_541_MOESM2_ESM.pdf]

Supplementary Information

Distinct brain morphometry patterns revealed by deep learning improve prediction of post-stroke aphasia severity

Alex Teghipco, Roger Newman-Norlund, Julius Fridriksson, Christopher Rorden, Leonardo Bonilha

Table of contents

Supplementary Methods	Pages 1-2
Supplementary Results	Pages 3-8
Supplementary Figure 1: Characterization of 19 studies retrieved from PubMed using CNNs and neuroimaging in stroke patients	Page 9
Supplementary Figure 2: Prevalence of different machine learning methods in neuroimaging more broadly and in neuroimaging of stroke patients	Page 10
Supplementary Figure 3: Comparing SVM models with different kernel functions and tuning methods	Page 11
Supplementary Figure 4: Additional approaches to stacking CNN and SVM models	Page 12
Supplementary Figure 5: Example feature saliency maps	Page 13
Supplementary Figure 6: SVM trained on CNN features	Page 14
Supplementary Figure 7: Ablation experiments	Pages 15
Supplementary Figure 8: Model-order selection for clustering Grad-CAM++ maps	Page 16
Supplementary Figure 9: Between and within-subgroup similarity	Page 17
Supplementary Table 1: Studies retrieved from PubMed using CNNs and neuroimaging in stroke patients	Page 18
Supplementary Table 2: Paired two-sample t-tests comparing feature saliency in severe and nonsevere patients within different regions of interest (ROIs)	Page 19
Supplementary Table 3: Lesion size and accuracy across severe patient subcategories	Page 20
Supplementary Table 4: Lesion size and accuracy across nonsevere patient subcategories	Page 21
Supplementary References	Page 22-23

Supplementary Methods

Deep learning

Due to the computational expense associated with repeating the nested cross-validation procedure we used, the number of possible hyperparameter values was generally restricted to ~3, representing, for example, relatively high vs low regularization. We approximated the magnitude of the learning rates to use for tuning by experimenting with a random split of the concatenated training folds (70% training, 30% validation) and used the shallowest CNN architecture to find the best performing model from among a larger set of learning rates (0.1, 0.001, 0.0001, 0.00001, 0.000001, 0.0000001).

A series of metrics were tracked to initiate early stopping and avoid overfitting. The minimum loss on the validation dataset was used to select the trained model for testing. As long as the model had attained a minimum F1 score of 0.7 on the validation set, training was ceased if: i) training data was predicted with greater than 94% accuracy, ii) validation data was predicted with an F1 score above 0.9, iii) loss on the validation set had not improved at least once over the last 50 epochs, and iv) training and validation F1 scores were inversely correlated over the prior 30 epochs for 50 epochs in a row (i.e., had a Pearson correlation coefficient below -0.3; F1 scores were used instead of loss because they appeared to more clearly indicate when the model began overfitting).

Ensemble clustering

Consensus matrices were used to assess the internal validity of clustering solutions by measuring their proportion of ambiguously clustered pairs (PAC)[1]. PAC serves to quantify the degree to which the cumulative distribution function (CDF) for consensus values across a consensus matrix displays a significant plateau in its middle portion. It is calculated by determining the percentage of pairings whose consensus indices fall within a specific range, typically [0.1, 0.9], which is the range we have used here, to highlight ambiguities in clustering. The core premise is that a clustering outcome demonstrating high reliability will yield a consensus matrix filled with values at the extremes—near zero and one—representing clear decision boundaries about whether pairs of samples should be grouped together or separated. Such distributions will result in a CDF with pronounced peaks close to the extremes and a notably flat section in between. PAC has robustly identified clustering solutions in well-characterized datasets, outperforming alternative but similar approaches grounded in evaluating clustering reliability (e.g., visual inspection of blockedness of heatmaps of the consensus matrix, or elbow in the change of area under the CFD curve when increasing the number of clusters)[1].

A notable limitation of PAC is its tendency to favor clustering models that generate a higher number of clusters[2]. We have observed that PAC generally follows expected patterns as the complexity of the solution (i.e., number of clusters) increases; however, it eventually begins to precipitously decline. This occurs because complex clustering solutions can become so fragmented that the consensus matrix almost uniformly reflects a lack of agreement about cluster assignments (i.e., is dominated by zeros with very few instances where samples are consistently clustered together). When inspecting a CDF curve for such a consensus matrix, it will have a significant peak at zero, a small peak near one, and a relatively flat mid-section as there is no ambiguity about cluster assignments. Thus, when consensus converges on agreement that most samples do not belong together, PAC can be misleadingly low.

To address this issue, we introduced a method that evaluates the overall consensus quality beyond just PAC by examining the degree to which consensus matrices exhibit a unimodal distribution. We employed Hartigan's dip test[3] to assess the unimodality of consensus matrices, calculating the deviation between the empirical distribution function and the closest unimodal distribution that minimizes this discrepancy. The significance of the unimodality, and thereby the likelihood of the consensus matrix being multimodal, was determined through a p-

value obtained by comparing the calculated dip value against values from 10,000 bootstrapped samples drawn from a uniform distribution. By disregarding solutions characterized by significantly unimodal consensus distributions, we were able to filter out solutions with overall poor consensus but low PAC.

Affinity propagation

Affinity propagation is an information-theory based clustering approach that can operate over similarity matrices and therefore can be applied to consensus matrices. In this clustering approach, two types of real-valued messages are exchanged between samples in order to group them by their most representative exemplar. That is, the algorithm explores the suitability of each data point to act as an exemplar, eventually converging on a set of exemplars that best represent the data.

During each cycle of affinity propagation, data points (samples) send responsibility messages to identify the most suitable exemplars, assessing each exemplar's ability to represent the sender effectively. Subsequently, availability messages are relayed back from these potential exemplars to the samples, compiling support for the fitness of each sample selecting the exemplar in question, considering the collective preferences of other samples. Initially, the values for both availabilities and responsibilities are set to zero for all sample pairs and are refined through successive iterations with the aid of a damping factor, which moderates the updates' impact. The calculation of responsibility from a sample i to a potential exemplar k involves subtracting the highest combined availability and similarity score of all other potential exemplars (excluding k) from the similarity score between i and k. Meanwhile, the availability from a potential exemplar k to a sample i is determined by taking the lesser of zero and the sum of k's self-assigned responsibility plus the total of positive responsibilities assigned to k by other samples, excluding i and k. After a series of iterations, where availabilities and responsibilities are periodically updated, the algorithm selects final exemplars for each sample based on the highest combined availability and responsibility.

Unlike traditional clustering methods, affinity propagation does not require a predefined number of clusters; instead, it employs a preference parameter, modified across the similarity matrix diagonal, to guide the selection of exemplars based on prior expectations. In our approach, we adjust the preference value iteratively, employing a bisection method to achieve a target number of clusters, thus incorporating a predefined cluster count into the affinity propagation algorithm's flexible framework[4].

ROI analysis of feature saliency

Perilesional ROIs were generated by applying mean dilation with a 3D box kernel of 10 mm to the lesion ROI, then masking the resulting map by the lesion ROI. Extralesional ROIs were created by including all voxels in the left hemisphere that were not contained in the lesion and perilesional ROIs. Homologue ROIs were generated by multiplying voxel coordinates in mm space by -1 (i.e., exploiting symmetry of the anatomical template).

Supplementary Results

CNNs in previous neuroimaging and stroke studies

The main text cites and characterizes 19 PubMed studies that use CNNs and neuroimaging in stroke patients. A more detailed categorization of these studies is presented in a Sankey diagram (Supplementary Figure 1) and the full list of studies can be found in Supplementary Table 1. Note, of the 19 studies, one was a review of stroke segmentation studies, one included stroke in its abstract but treated Alzheimer's patients, and one attempted identification of silent strokes from natural language processing of patient notes. In addition, 2 studies were focused on improving acquisition sequences with CNNs. Many of the studies for which the main goal was either identification or segmentation were not in stroke patients. These studies treated intracranial hemorrhages, arterial brain vessels, and microbleeds. A total of 13 studies treated stroke patients.

t-SNE

We sought further evidence for CNN model robustness by verifying that it was able to learn consistent features across repeats of the cross-validation procedure. Feature consistency indicates the model is stable and can sufficiently capture underlying patterns without overfitting to the idiosyncrasies of specific training sets or fitting to noise or artifacts that may be present in the data. To this end, t-distributed stochastic neighbor embedding (t-SNE) was used to find a low-dimensional visualization of the features learned across all CNN models. Specifically, we combined models from the outer folds of all 20 cross-validation repeats and extracted their first fully connected layer activations, which act to map high-level features learned by the CNN on to a slightly lower dimensional representation for separating classes. If the CNNs had learned meaningful and consistent features, we expected to see class separation in the embedding. An optimal perplexity value for t-SNE was selected by evaluating solution divergence (Kullback-Leiber) across a large range of perplexity values. Generally, divergence decreases with perplexity so when this criterion is used for model selection, the highest tested perplexity value tends to be selected, favoring the most complex models. We weighted divergence by the magnitude of perplexity and used the pseudo BIC index to find the lowest perplexity value that minimized divergence, helping to balance t-SNE model complexity and accurate representation of the CNN features in an objective way (see[5] and https://github.com/alexteghipco/evalTSNE for implementation). Figure 3c (main text) shows clear and distinct clusters for different class predictions in the t-SNE embedding, confirming that the features learned by CNNs have a structure that exhibits consistency across repeats of cross validation. As t-SNE embedded samples based on an ensemble of CNN features, we determined the sample predictions of the CNN ensemble by taking the median prediction across all models (i.e., to verify that sample clusters in the embedding mapped onto class predictions). Class separation in the embedding was weaker when considering true class labels instead of predicted labels, however, this fundamentally reflected the models' tendencies to trade-off more false positives for better severe aphasia prediction accuracy.

Comparing linear and nonlinear SVMs

Our comparisons between classical machine learning and deep learning used an optimistically biased estimate of SVM performance. This provided relatively more compelling evidence that SVMs underperformed compared to CNNs. An optimistic bound for SVM performance was estimated by training separate SVM models using linear and nonlinear kernel functions and retaining the best performing models for comparison to CNNs (see methods from main text). Here, we show that tuning the kernel function alongside other hyperparameters when building SVM models results in substantially worse performance than simply tuning the same parameters but holding the kernel function fixed to be a linear SVM (Supplementary Figure 3).

This was the case whether the SVM was trained on all features in the data, or if dimensionality reduction was applied during training. When linear and nonlinear SVMs were trained independently, linear SVMs tended to outperform nonlinear SVMs. Note, performance for SVMs that were tuned for kernel function and did not involve dimensionality reduction as a preprocessing step was measured over 10 instead of 20 repeats of our cross-validation scheme (these models took the longest to train). However, the distribution of performance suggests a relatively consistent peak around 0.5 for F1 scores.

Evaluating the quality of CNN model predictions against classical machine learning

SVMs generated substantially worse severe class accuracies (M=0.7, SD=0.04) than CNNs (M=0.88, SD=0.03), $t(19) = -15.73$, $p < 0.00000001$, Cohen's $d = -3.52$. However, they had higher nonsevere accuracy (SVMs: M=0.76, SD=0.02; CNNs: M=0.66, SD=0.03), $t(19) = 12.34$, $p < 0.00000001$, Cohen's $d = 2.76$. Although SVMs did achieve significantly higher precision (M=0.61, SD=0.01) than CNNs (M=0.58, SD=0.01), $t(19) = -6$, $p < 0.00001$, Cohen's $d = 1.36$, CNN outperformance on F1 and mean accuracy measures was associated with larger effect sizes as reported in the main text.

When dimensionality reduction was introduced, SVMs performed slightly worse on precision, $t(19) = -5$, $p < 0.0001$, Cohen's $d = -1.1$ (SVM: M=0.61, SD=0.01; SVM with dimensionality reduction: M=0.58, SD=0.03) and exhibited lower nonsevere class accuracy, $t(19) = -5.5$, $p < 0.0001$, Cohen's $d = -1.22$ (SVM: M=0.76, SD=0.02; SVM with dimensionality reduction: M=0.71, SD=0.02).

Supplemental approaches to stacking CNN and SVM models

We report the results for stacking with LDA but note that, in our experiments (i.e., qualitative inspection of median performance measures across folds in one repeat of cross-validation), logistic regression and decision trees appeared to perform just as well. We also tested whether stacking would improve if it additionally had access to the features the lower-level models were trained on (i.e., combining lower-level model predictions and all brain features in the data). Inputs used to train lower-level models were included as inputs to the stacked model from Figure 5b. Just as in the analysis presented in the main text, here we trained and tested the stacked model over a range of hyperparameters (see Figure 5b for more information about range). The best-case performing stacked models from the main text and this analysis are compared in Supplementary Figure 4. Exposing the stacked model to the inputs of the lower-level models resulted in worse performance on the F1-score.

Although stacking did not produce significantly better predictions (Figure 5), it is possible that classical machine learning can improve overall model accuracy by more successfully exploiting features learned by the CNN. Further, it may be the case that PCA and ICA did not boost the performance of SVMs because the lower dimensional spaces produced by these methods did not capture the data as accurately (i.e., poorer SVM performance would still be attributable to the dimensionality of the data). If that were the case, we would expect SVMs to perform at least as well as CNNs when trained on CNN feature maps**.** Our results indicate this to be the case (Supplementary Figure 6). Training SVMs on the CNN features embedded in the fully connected layers of the networks improves model performance relative to using dimensionality reduction (PCA/ICA) and results in SVMs that are roughly as good as CNNs at predicting aphasia severity. This in turn clarifies that SVMs were not better at exploiting the features learned by the networks. SVM with CNN features (M=0.69, SD=0.02) performed substantially better than SVM with dimensionality reduction (M=0.65, SD=0.02) based on F1 scores, $t(19) = 8.8$, $p < 0.0000001$, Cohen's $d = 2.0204$. This was also the case for mean accuracy (SVM with CNN features: M=0.77, SD=0.02; SVM with dimensionality reduction (M=0.72, SD=0.02), $t(19) = 8.6$, $p < 0.0000001$, Cohen's $d = 2$. Moreover, there was no significant difference between F1 scores for CNN models and SVM models trained on CNN

features ($p = 0.2$). There was also no significant difference between these models for mean accuracy ($p = 0.24$). However, SVMs trained with CNN features (M=0.7, SD=0.03) attained higher nonsevere class accuracy than CNNs (M=0.66, SD=0.03), $t(19) = 6.4$, $p < 0.00001$, Cohen's d = 1.5 but lower severe class accuracy than CNNs (SVMs with CNN features: M=0.83, SD=0.04; CNNs: M=0.88, SD=0.03), $t(19) = -5.72$, $p < 0.0001$, Cohen's d = 1.3. SVMs trained with CNN features (M=0.6, SD=0.02) also attained higher precision scores than CNNs (M=0.58, SD=0.02), $t(19) = 5.2$, $p < 0.00001$, Cohen's d = 1.2. As SVMs trained on CNN features did not improve the F1 score and decreased severe prediction accuracy, we interpret these results to suggest SVMs do not meaningfully improve on the CNN model. SVM models trained on CNN features did not perform significantly better than SVM models trained on Grad-CAM++ maps (c.f., Figure 7) according to F1 scores, $t(19) = 1.52$, $p = 0.15$, Cohen's d = 0.39.

Ablation experiments

We verified that interhemispheric relationships in the Grad-CAM++ features were meaningful (e.g., homologues of the lesioned and perilesional area were often highlighted) by training SVMs independently on just the left and right hemisphere portions of the saliency maps (Supplementary Figure 7a). We found that using bilateral features resulted in higher F1 scores (M=0.69, SD=0.02) than restricting the analysis to right hemisphere features (M=0.66, SD=0.04), $t(19) = 5.15$, $p < 0.0001$, Cohen's d = 1.2. Bilateral features also resulted in higher F1 scores (M=0.69, SD=0.02) compared to left hemisphere features (M=0.67, SD=0.04), $t(19) = 3.1$, $p < 0.01$, Cohen's d = 0.7.

A direct confirmation that features outside of the lesion drove model performance was achieved by rerunning the CNN model training, tuning, and testing on the same exact partitions of our cross-validation while removing any non-lesion related information in the data. The lesion-only CNN (M = 0.68, SD = 0.024) performed significantly worse on the F1 score than the CNN model that was trained on all features (M=0.7, SD=0.01), $t(19) = -2.45$, $p < 0.05$, Cohen's d =-0.7 (Supplementary Figure 7b). The magnitude of this effect is generally in line with our interpretation in comparing CNN and SVM performance—that the spatially dependent patterns picked out by the CNN that chiefly highlight morphometry in the right hemisphere provide an incremental improvement on model accuracy.

Individual-level SHAP maps

We expected deep SHAP maps generated for CNN and SHAP maps generated for SVM to share similarities, and to highlight very different trends compared to Grad-CAM++ maps generated for CNN. This finding would be consistent with the fact that Grad-CAM++ alone can recognize spatial dependencies exploited by the CNN. Supplementary Figure 5 qualitatively illustrates this to be the case in a random sample of 6 participants that were correctly predicted by the CNN to have severe aphasia, and another 6 participants that were correctly predicted by the CNN to have nonsevere aphasia. Saliency maps were scaled for cleaner comparison of magnitude and relative importance. Note that Grad-CAM++ maps only represent feature importance for the predicted class while SHAP and deep SHAP maps represent feature importance that drives the model towards the predicted class (positive SHAP values) as well as the opposite class (negative SHAP values). SHAP maps were scaled between -1 and 1 instead of 0 and 1 to preserve this signed information.

Across patients with different lesion sizes, the feature maps clearly demonstrated that Grad-CAM++ attributes successful prediction of severe aphasia by CNNs to features in the contralateral hemisphere, and attributes nonsevere prediction to ipsilateral features. Sometimes ipsilateral and contralateral features that were highlighted included the lesion, the perilesional area or their homologues. Although deep SHAP maps also explained CNN model predictions, they showed a vastly different spatial pattern, with both successful prediction of severe and nonsevere aphasia being attributed to features encompassing the lesion. Features that pulled

predictions towards the opposite class appeared to highlight the intact portions of the perisylvian cortex, where lesion overlap across the cohort happened to be concentrated (see Figure 6 in main text). This suggested deep SHAP maps emphasized one of the multiple patterns exploited by the CNN: lesion size. SVM SHAP maps overall looked very similar to deep SHAP but severe aphasia predictions emphasized peripheral intralesional features whereas nonsevere aphasia predictions emphasized perilesional features. This more complex pattern implied that like the CNN, the SVM did not make predictions strictly based on lesion size. However, overall, it was much clearer from the feature maps that the CNN exploited information outside the lesion.

Subtyping patterns learned by the CNN

Patients were clustered into subgroups with similar Grad-CAM++ maps using consensus clustering. A consensus matrix was generated for clustering solutions ranging from 3 to 30 clusters. The distribution of consensus values within each of these matrices are presented in Supplementary Figure 8a and were used to guide model-order selection, the aim of which was to identify the most complex solution that remained reliable or stable, with high consensus across clustered subsamples of the dataset. Reliable solutions should be disproportionately made up of consensus values around 0 and 1, reflecting the fact that clustering always placed pairs of samples into the same or different clusters. The proportion of ambiguously clustered pairs (PAC) was used to assign each of these distributions a gross “reliability” value (see methods from supplemental material and main text) by capturing the flatness of a cumulative distribution function of consensus values within a consensus matrix. PAC across the range of investigated clustering solutions is presented in Figure Supplementary Figure 8b. PAC can be misleading when clustering mainly places pairs of samples into different clusters (i.e., shows mostly consensus values of 0 and not many consensus values of 1). Such solutions were eliminated from consideration by testing whether consensus distributions were significantly unimodal using Hartigan’s dip test (see opaque area in Figure Supplementary Figure 8b). For severe patients, we selected the solution with the overall lowest PAC (i.e., 0.000015), which contained 7 clusters. For nonsevere patients, we selected the solution with the 3rd highest PAC, which was still remarkably low (0.005). Put another way, the selected solution showed ambiguity in cluster assignment for only 0.5% of samples in the data. Affinity propagation was used to extract the final clustering from the selected consensus matrices. We verified that affinity propagation could readily identify the structure of consensus matrices by repeating the clustering process 1000 times. We note that clusters were identical every single time affinity propagation was repeated. We also report that the exemplar samples around which affinity propagation organized clusters was highly consistent (Supplementary Figure 8c). For severe patients, exemplars occasionally shifted between repeats of affinity propagation for some clusters. However, there was always a clear sample (i.e., patient) around which clusters formed across the majority of repeats.

Pearson correlation coefficients were estimated between Grad-CAM++ maps and sorted by patient subcategory to illustrate the extensive degree of within-subgroup similarity and between-subgroup dissimilarity. Further, correlation coefficients were divided into those measuring similarity between all patients within a subgroup, and those measuring similarity between patients within a subgroup and patients of other subgroups. The means of these correlation coefficients were used to probe within-subgroup and between-subgroup similarities (Supplementary Figure 9).

Clustering revealed more diverse brain integrity patterns being exploited by the CNN than suggested by the relatively localized group-averaged saliency effects (Figure 9a; note all figure references in this section are for the main text). Consistent with the observation that left hemisphere regions still exhibited moderate feature importance (Figure 8), some severe aphasia patient subgroups showed saliency that peaked in the right hemisphere but was still relatively high in the left hemisphere (Figure 9b). As anticipated by the group map, most

subgroups showed a strong peak in right anterior frontal cortex (subgroups 1,2,3,7). This peak was generally concentrated in a region spanning right middle frontal gyrus (MFG) and inferior frontal gyrus (IFG) (subgroups 1,2), however, some subgroups exhibited a peak in only one of these regions (MFG:7, IFG:3). One subgroup showed concomitant high saliency in the right temporal pole (TP) and slightly lower saliency in left MFG (subgroup 3). In contrast, other patients showed relatively high saliency in right IFG but had a stronger peak in right TP (subgroups 5 and 6). Patients showing this pattern could be subdivided further based on whether they additionally exhibited relatively higher feature importance in left parietal cortex (subgroup 5). The patient subgroups where feature saliency peaked in a region spanning right MFG and IFG could also be subdivided based on whether features in the left hemisphere were highlighted (subgroup 1), and whether the right posterior inferior temporal cortex (pITC) had moderately high importance (subgroup 2). Finally, one patient subgroup emphasized right occipital cortex (OC) but showed some feature importance in left anterior frontal and temporal cortex (subgroup 4). Notably, the patterns indicated by the exemplar participant around which each subgroup formed were remarkably similar to the other participants within that subgroup, corroborating the analysis of cluster consensus (Figure 9c).

To further understand the extent to which each subgroup reflected altered morphometry patterns along different brain systems, we decoded subgroup saliency maps as typified by the exemplar (see methods for more details). Decoding (Figure 9c) confirmed that most feature saliency patterns reflected language function (i.e., subgroups 2, 4, 5, and 7) but also underlined the extent to which they tapped into different language subsystems. For example, one subgroup showing more focal saliency around the right TP and left superior parietal cortex (SPC) was associated with semantics (i.e., top 3 terms mapping onto topic: semantic, word, knowledge; subgroup 5), whereas another subgroup (i.e., 2) showing saliency along large portions of frontal cortex and inferior temporal cortex (ITC) was associated with lexical-semantics, reading, comprehension, and pitch processing (i.e., topics: reading, phonological, readers; speech, auditory, temporal; words, word, lexical; verbs, verb, noun; language, sentences, comprehension; music, musical, pitch). Further, the subgroup with peak saliency in right OC (subgroup 4) displayed strongest associations to language, including reading and lexical processing (topics: hearing, deaf, sign; words, word, lexical; reading, phonological, readers; semantic, word, knowledge), but also tool knowledge (topic: tool, tools, knowledge) and sensory cortex (i.e., topic: visual, auditory, sensory). At the same, this subgroup displayed saliency associated with selective adaptation (topic: adaptation, selective, stimulus), as well as motion and object processing (topics: motion, mt, moving; object, objects, visual). A similar pattern of topics was associated with the subgroup exhibiting peak saliency around right MFG (i.e., subgroup 7), but also included associations to comprehension (i.e., topic: language, sentences, comprehension) and overt spontaneous speech (topic: verbal, fluency, overt). This subgroup was also associated with the dorsal and ventral streams despite being relatively focal (i.e., topic: dorsal, ventral, stream), as well as task switching, integration, item pairs, and action observation (topics: integration, process, task and task, switching, set; item, pairs, item; action, actions, observation). Curiously, several subgroups exhibited saliency related to other studies on morphometry (topic: matter, gray, volume; subgroups 6) as well as age (i.e., topic: adolescents, adolescent, age; subgroups 1, 3, 6). These particular subgroups were also associated with a large number of topics representing demographics (e.g., sex), higher-level cognitive functions (e.g., decision making and social interactions), and a number of disorders (e.g., PTSD, AD, OCD, depression, epilepsy, ADHD, substance abuse, generic symptom severity, etc).

Despite diffuse feature importance observed at the group-level (Figure 10a), clustering of patients with nonsevere aphasia also highlighted diverse patterns of brain integrity learned by the CNN. Combined with the results of Figure 9, these patterns demonstrated that irrespective of aphasia severity, CNN predictions tended to revolve around the same anatomical regions, configured into slightly different networks, and with the hemisphere driving prediction generally

varying as a function of severity. For example, a subgroup of patients with severe aphasia displayed highest feature saliency in right OC, while a subgroup of patients with nonsevere aphasia displayed peak saliency in left OC, but to a lesser extent also implicated portions of right OC (subgroup 4). A similar pattern, whereby the right hemisphere of a comparable network was emphasized for nonsevere patients, can be observed for a subgroup with feature saliency peaking in the TP and anterior frontal cortex (e.g., IFG; subgroup 2). Further, at least one saliency pattern indicated a shared network among aphasia subtypes, exhibiting peak saliency in right ITC but also high saliency in right MFG (subgroup 6). For patients with nonsevere aphasia, this network differed by having shifted distributions of peak saliency, showing relatively higher saliency in right ITC than MFG and additionally exhibited modest saliency in left MFG. Other subgroups had saliency maps that peaked around: i) just the left anterior TP, without additional peaks in anterior frontal cortex as observed for other subgroups (subgroup 1), ii) a region of the left SPC with slightly lower saliency extending anteriorly into motor cortex (subgroup 3), and iii) the same region of left SPC but with slightly lower saliency extending inferiorly into inferior parietal cortex (IPC) (subgroup 5). In multiple subgroups, right frontal (subgroup 1,6), right temporal (subgroup 2, 4,6) and right occipital cortex (subgroup 4) showed relatively moderate feature importance. Note, the subgroup that showed strongest right hemisphere lateralization of saliency exhibited high accuracy (Supplementary Table 2).

Decoding of patient subtypes (Figure 10c) demonstrated that most patterns of morphometry associated with nonsevere aphasia were unrelated to language. Although many patients with nonsevere aphasia were grouped into subtypes that displayed peak saliency in regions commonly associated with language, only one subgroup (i.e., 6) showed a decoding pattern that mapped strongly onto language function (topics: semantic, word, knowledge; word, words, lexical). Coincidentally, this was the only subgroup exhibiting a right-lateralized saliency pattern. The saliency patterns of two subgroups (i.e., 1 and 2) were associated with morphometry studies (topics: matter, gray, volume) and one of these subgroups also displayed an association with age (topic: adolescents, adolescent, age; subgroups 2). Consistent to patients with severe aphasia that had similar topic associations, decoding also showed a strong relationship to demographics (e.g., sex), higher-level cognitive functions (e.g., emotional regulation, emotional processing, social interactions) and a number of disorders (e.g., PTSD, AD, OCD, depression, epilepsy, ADHD, substance abuse, schizophrenia, anxiety, BPD, generic symptom severity, etc.). This similarity between severe and nonsevere patient decodings is unsurprising, reflecting overlapping patterns of saliency despite different lateralizations The subgroup that demonstrated peak saliency in left OC (i.e., 4) decoded in a similar manner to the severe patient subgroup with peak saliency in right OC. Strong associations were observed with motion and object processing as well as visual processing more generally (topics: motion, mt, moving; object, objects, visual; blind, sighted, judgments; face, faces, fusiform; orientation, colour, separation; visual, auditory, sensory; spatial, location, space; perceptual, perception, visual; color, shape, shapes). The two subgroups with peak saliency favoring different aspects of SPC (i.e., subgroups 3 and 5) demonstrated somewhat different decoding patterns, although both were strongly associated with spatial attention, working memory and reaction time (topics: spatial, location, space; attention, attention, visual; eye, gaze, saccade; wm, load, memory; response, time, reaction). The more anterior saliency pattern was uniquely associated with inhibition (topics: inhibition, response, inhibitory) while the posterior saliency pattern exhibited a broader decoding to unique topics. These included numerical processing, motion, imagery, hand movement, object recognition, and task training (topics: motion, mt, moving; imagery, mental, rotation; color, shapes, shape; ips, number, numerical; object, objects, visual; hand, movements, limb; bling, sighted, judgments; action, actions, observation, orientation, color, separation; movement, motor, movements; training, practice, trained, etc).

Supplementary Figures

Supplementary Figure 1. *Characterization of 19 studies retrieved from PubMed using CNNs and neuroimaging in stroke patients. From left to right, studies are categorized by year of publication (dark blue), their broad purpose for using a CNN (e.g., the CNN is used for segmentation; light blue), the specific subject to which they are applying the CNN (e.g., stroke patients; green), and the specific target the CNN is used to predict (e.g., lesions; yellow).*

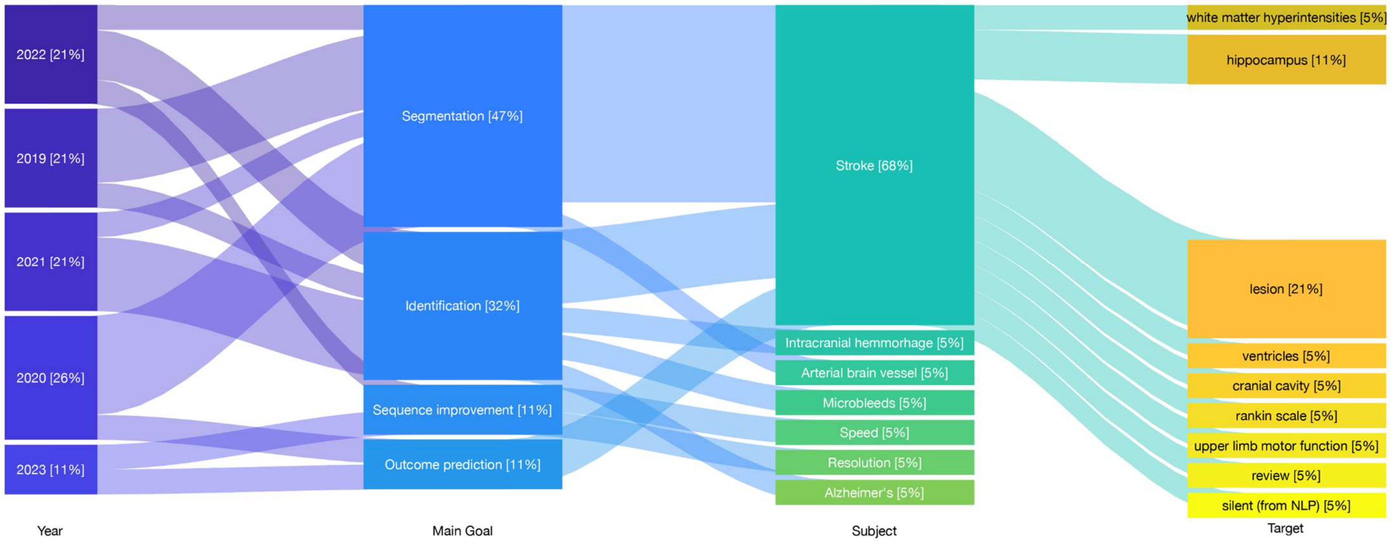

Supplementary Figure 2. *Prevalence of different machine learning methods in neuroimaging more broadly (left) and in neuroimaging of stroke patients (right). The number of publications using different machine learning methods were retrieved from PubMed (y-axis) with the search query neuroimaging [AND] the method specified in the legend. In the panel on the right an additional phrase was added to the search: [AND] stroke. Both plots show that while support vector machines remain the most prevalent machine learning approach in studies, convolutional neural networks (CNNs) are becoming increasingly more frequently used and oftentimes are used more frequently than other classical machine learning methods, which include bagging and boosting of weaker learners in general, bagging of decision trees specifically (i.e., Random Forest), and linear regression with L1 (LASSO) or L1 and L2-norm penalties (Elastic Net).*

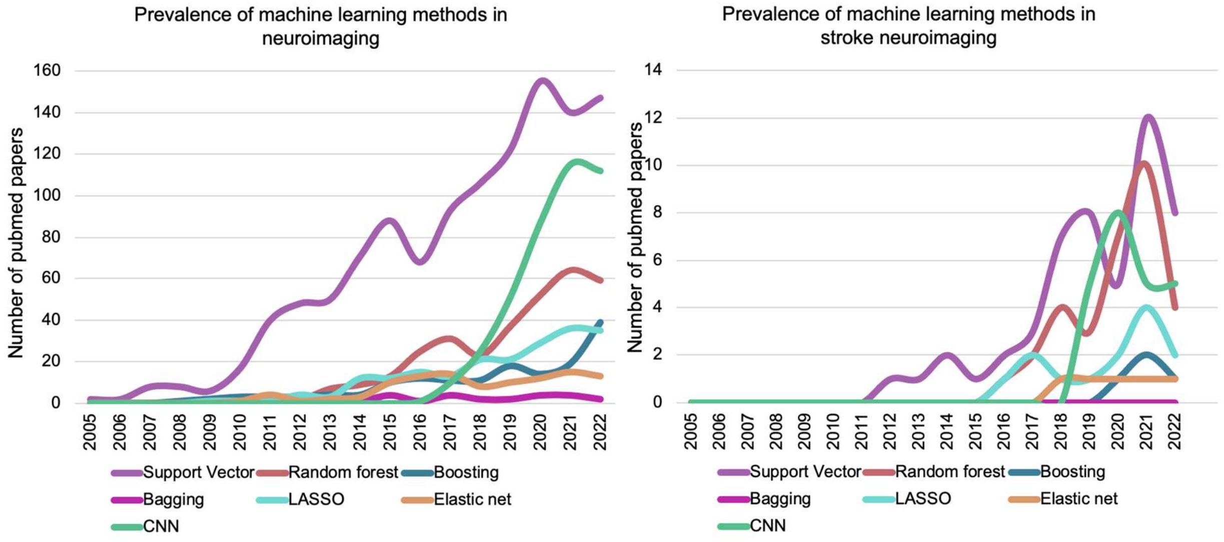

Supplementary Figure 3. *Comparing SVM models with different kernel functions and tuning methods. The bottom two violin plots show data from the main text, reflecting SVM performance with a fixed linear kernel. In the bottom blue plot dimensionality reduction is not employed but in the purple plot above it, ICA is used. Including kernel function (i.e., linear and radial basis functions) as a hyperparameter lowers model performance. A SVM without dimensionality reduction performs worse when the kernel function is tuned (pink). A SVM with dimensionality reduction also performs relatively worse when the kernel function is tuned (orange). Finally, fixing the SVM to have a radial basis function kernel (nonlinear) also lowers SVM performance when dimensionality reduction is not used (yellow).*

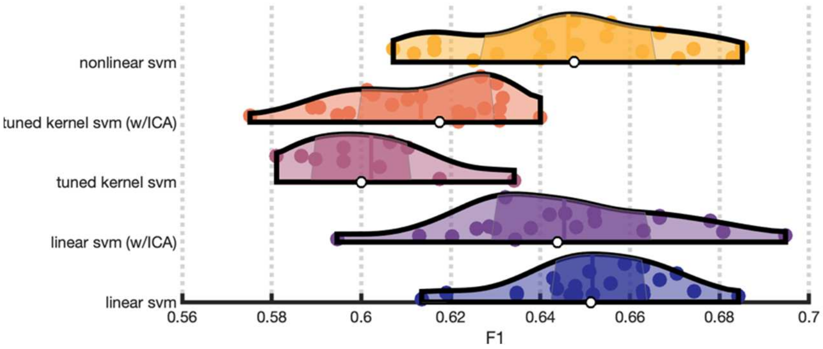

***Supplementary Figure 4**. Additional approaches to stacking CNN and SVM models. Performance of a stacked model trained on all brain features and the predictions of lower-level SVM and CNN models is displayed on top (salmon). Performance of a stacked model trained only on brain features is displayed on bottom (purple).*

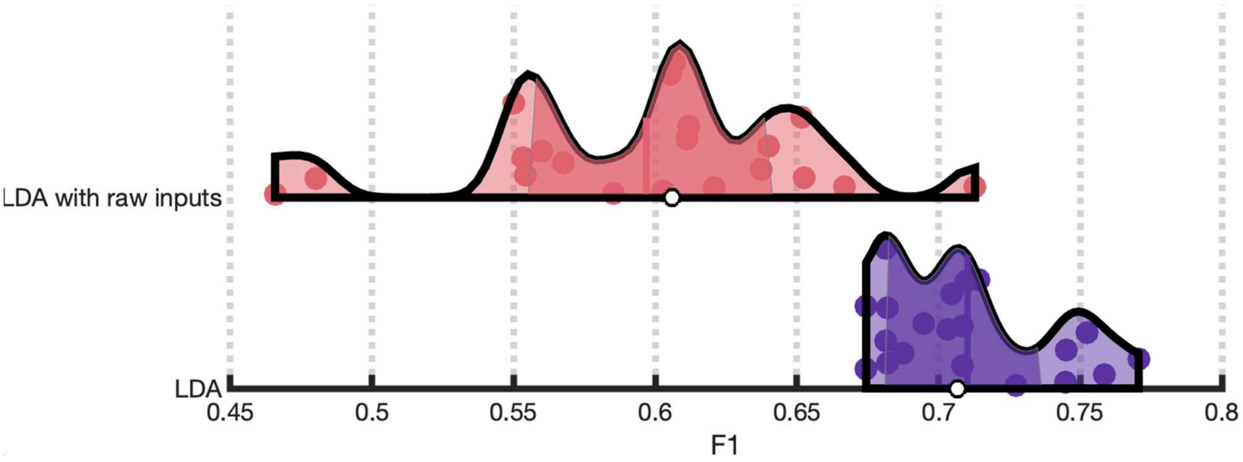

***Supplementary Figure 5.** Example feature saliency maps. Saliency maps that qualitatively highlight decision tendencies for the CNN and SVM models (i.e., across repeats). Each panel shows a montage of upsampled feature saliency maps (blue to red solid colors) and lesion masks (pink outline) overlayed on a normalized template (black outlines). Panels a, b and c show the same six example patients with severe aphasia and panels d, e and f show the same six example patients with nonsevere aphasia. Subjects with small, medium, and large lesions were randomly selected from a pool of samples correctly predicted by the CNN. Panels a, b, s, and E show saliency maps for the CNN. Panels c and f show saliency maps for SVM. Within each panel, rows as separated by a horizontal line represent unique example subjects. **Panel a:** Montage of upsampled Grad-CAM++ saliency maps for correct severe aphasia predictions by CNN. Hotter solid colors represent relatively higher feature importance and cooler solid colors represent relatively lower feature importance for the prediction (see colorbar for panel d). **Panel b:** Montage of upsampled Deep SHAP saliency maps for correct severe aphasia predictions by CNN. Hotter solid colors represent relatively higher feature importance for the (correct) class prediction and cooler solid colors represent relatively higher feature importance for the opposite (incorrect) class prediction (see colorbar for panel e). **Panel c:** Montage of upsampled SHAP saliency maps for correct severe aphasia predictions by SVM. Color coding of feature importance is consistent with panel b (also see colorbar for panel f). Note, the SVM made an incorrect prediction for the second participant, explaining the reverse spatial pattern. **Panel d:** Montage of upsampled Grad-CAM++ saliency maps for correct nonsevere aphasia predictions by CNN. Color coding of feature importance is consistent with the other panels. **Panel e:** Montage of upsampled Deep SHAP saliency maps for correct nonsevere aphasia predictions by CNN. Color coding of feature importance is consistent with the other panels. **Panel f:** Montage of upsampled SHAP saliency maps for correct nonsevere aphasia predictions by SVM. Color coding of feature importance is consistent with the other panels.*

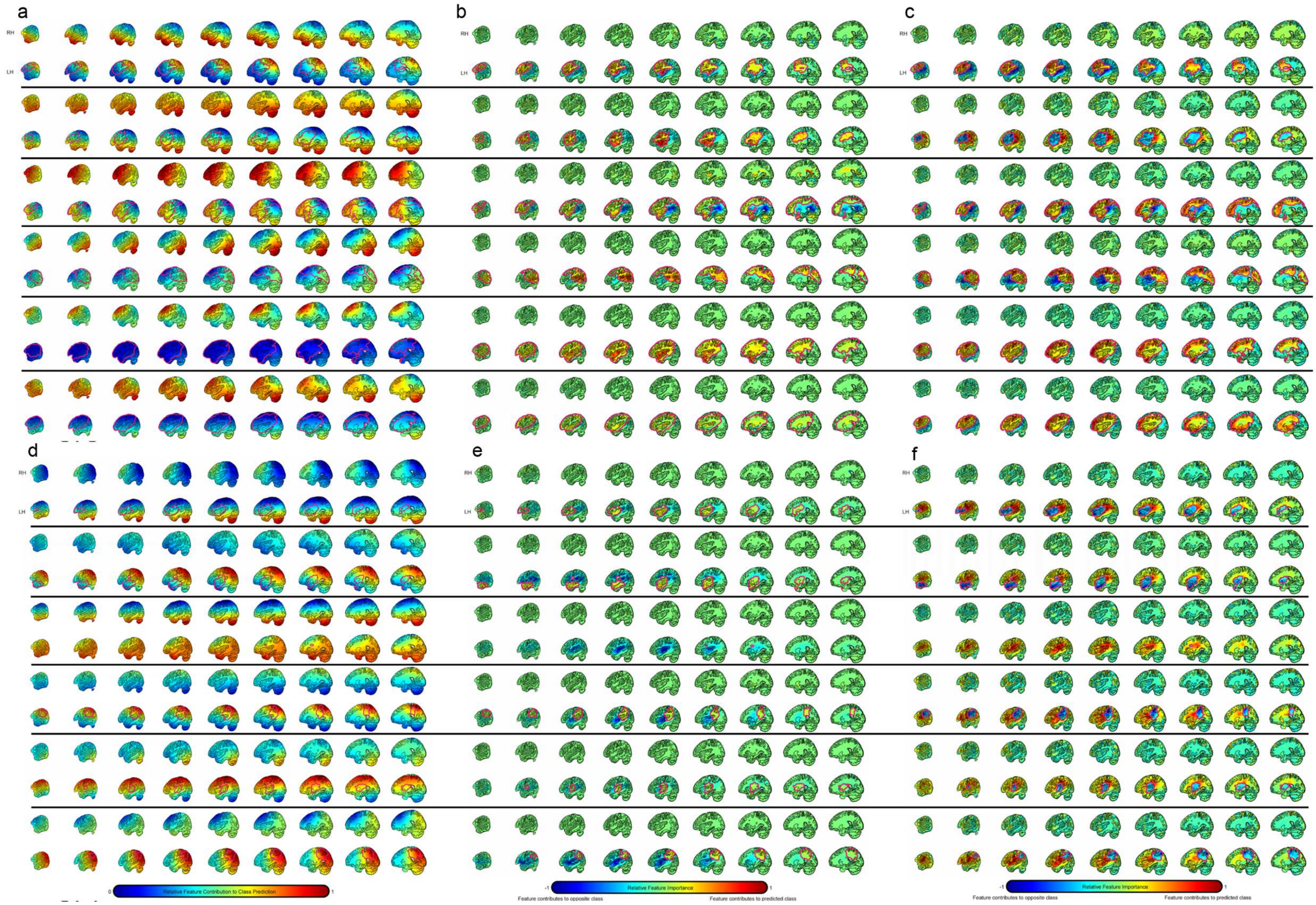
a
b
c
d
e
f
RH
LH
0
Relative Feature Contribution to Class Prediction
1
-1
Relative Feature Importance
Feature contributes to opposite class
Feature contributes to predicted class

Supplementary Figure 6. *SVM trained on CNN features. Violin plots showing CNN performance over 20 repeats of the cross-validation scheme relative to other strategies for fusing SVM and CNN predictions. Violin plot colors correspond to different performance measures which are additionally separated by horizontal lines. Within each performance measure the first or topmost violin plot shows the performance of a SVM trained on features extracted by a CNN (i.e., chaining CNN for feature extraction and SVM for prediction). The bottom violin plot shows CNN model performance as a baseline.*

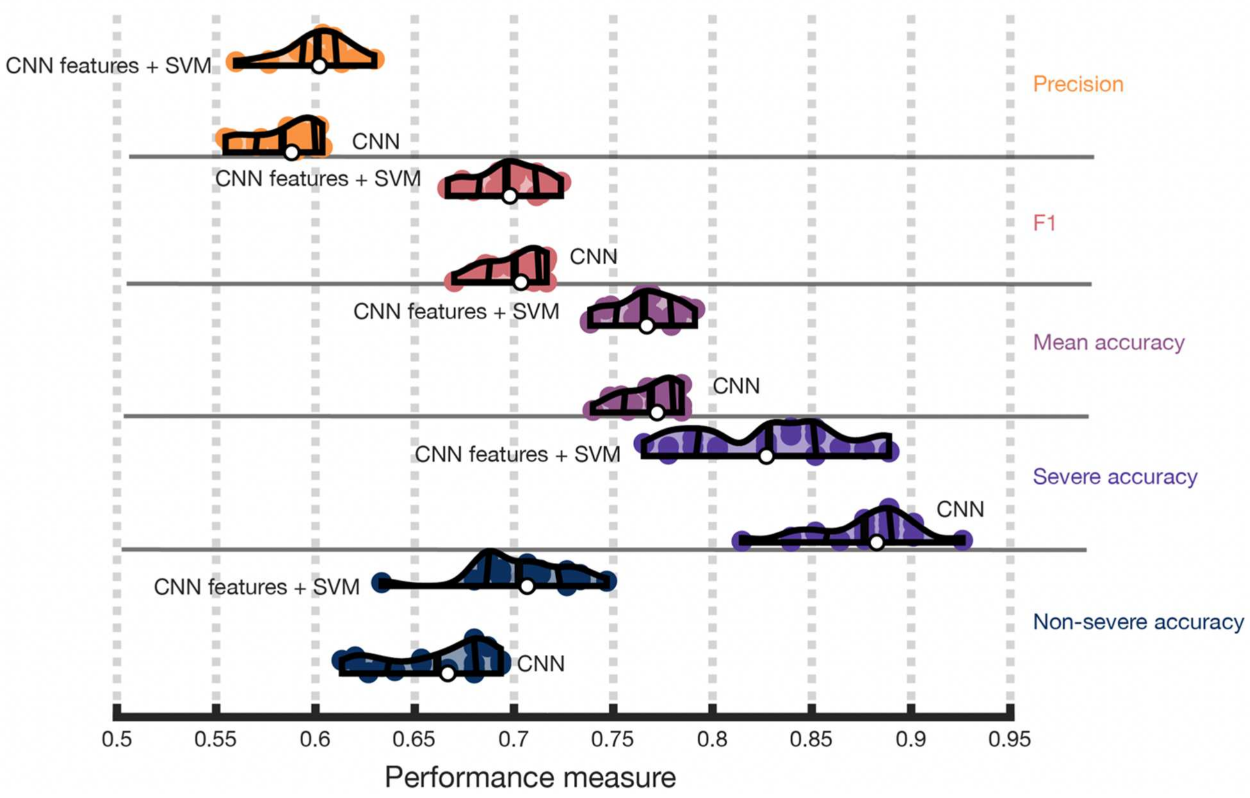

Supplementary Figure 7. *Ablation experiments.* ***Panel a:*** *Violin plots showing that a SVM trained on entire Grad-CAM++ saliency maps (orange) outperforms SVMs trained on just the left (yellow) or right hemisphere (pink) portions of the Grad-CAM++ maps according to the F1 score (x-axis).* ***Panel b:*** *Violin plots showing that a CNN trained only on lesion features (purple) performs worse than a CNN trained on all features (blue). An SVM model trained on all features is shown for comparison (pink).*

a: SVM-based ablation experiments using CNN saliency map

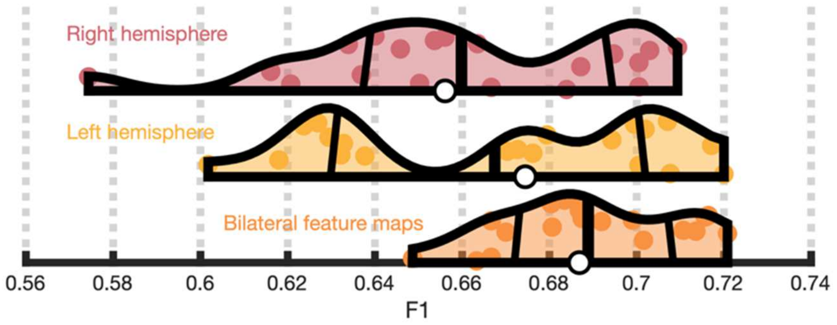

b: CNN-based ablation experiments

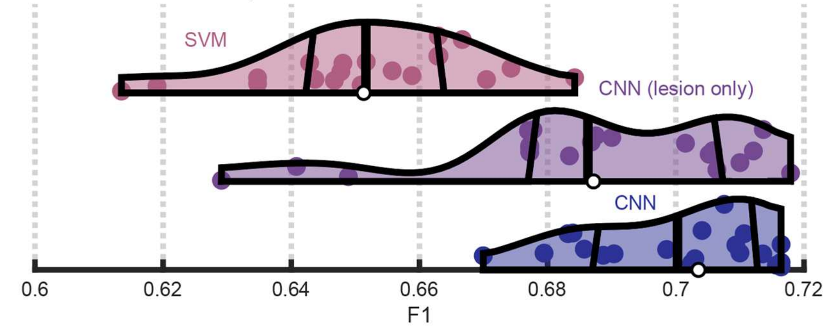

Supplementary Figure 8. *Model-order selection for clustering Grad-CAM++ maps. Data for severe patients are shown in the top plot of each panel, and data for nonsevere patients are shown in the bottom plot.* ***Panel a:*** *Consensus matrices are plotted as distributions for solutions containing between 3 and 30 clusters. Each solution is assigned a unique color. The solution that was eventually selected is highlighted by a dotted line. Consensus values being plotted reflect the proportion of times a sample was assigned to the same cluster across subsamples of the data.* ***Panel b:*** *An empirical cumulative distribution function was generated for each consensus distribution from panel a. The difference in this function at consensus values of 0.9 and 0.1 were taken to generate the Proportion of Ambiguously Clustered Pairs (PAC). PAC values are plotted as a function of increasing solution complexity (i.e., higher number of clusters in the solution). An opaque area highlights distributions that were significantly unimodal according to Hartigan's dip test, and therefore were eliminated from consideration during model order selection. The selected solutions are highlighted by colored dots corresponding to the legend from panel a.* ***Panel c:*** *Exemplars for affinity propagation (AP), applied over the selected solutions' consensus matrix, were tracked over 1000 repeats of the AP clustering process. The proportion of times a certain sample was selected as the exemplar is shown on the y-axis and individual samples are shown on the x-axis, colored according to the cluster to which they were assigned by AP (cluster assignment was unanimous across repeats).*

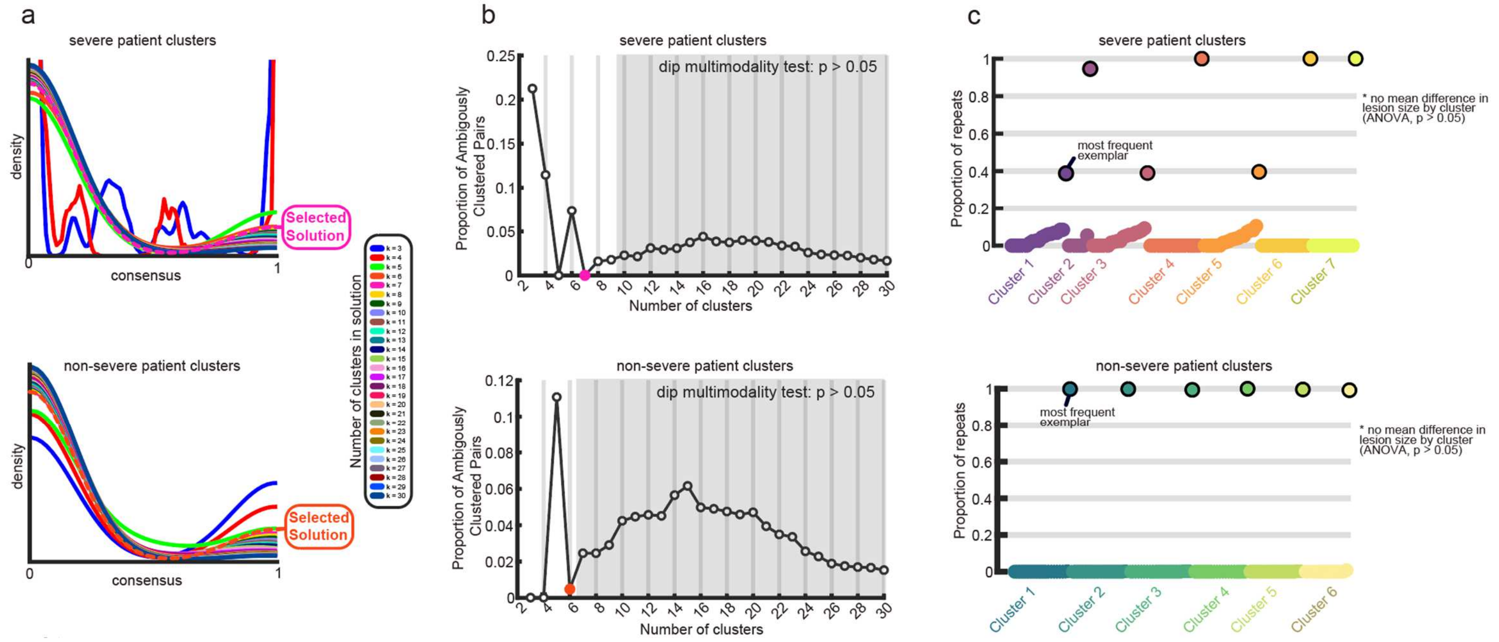

Supplementary Figure 9. *Between and within-subgroup similarity.* ***Panel a:*** *Pearson correlation coefficients were computed between all patients' feature saliency maps, then patients were sorted by subgroup (i.e., clusters).* ***Panel b:*** *Mean correlation coefficients were computed within-subgroups and between-subgroups for severe patients.* ***Panel c****: Identical to panel A but mean correlation coefficients were computed for nonsevere patients.*

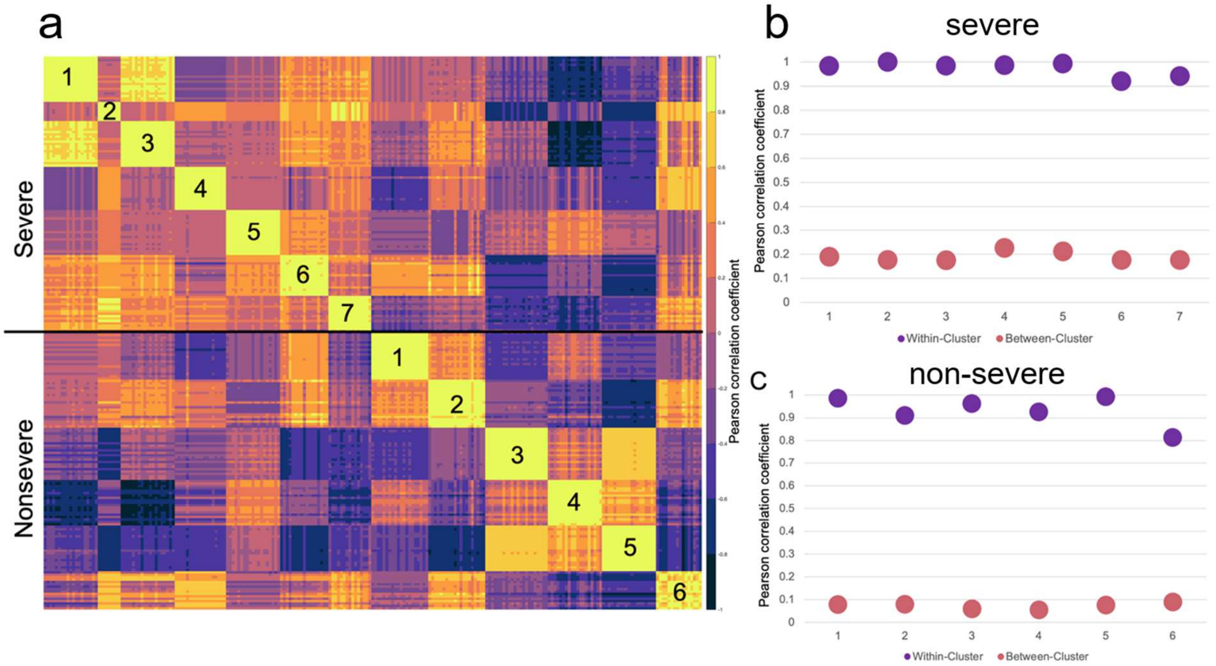

Supplementary Tables

Supplementary Table 1: Studies retrieved from PubMed using CNNs and neuroimaging in stroke patients.

Study	Year	Main goal	Subject	Specific target
Forooshani et al.[6]	2022	Segmentation	Stroke	white matter hyperintensities
Goubran et al.[7]	2019	Segmentation	Stroke	hippocampus
Heit et al. [8]	2021	Identification	Intracranial hemorrhage	Intracranial hemorrhage
Hilbert et al. [9]	2020	Segmentation	Arterial brain vessel	Arterial brain vessel
Li et al. [10]	2021	Identification	Microbleeds	Microbleeds
Meier et al. [11]	2019	Segmentation	Stroke	lesion
Nitiri et al. [12]	2021	Segmentation	Stroke	ventricles
Sheth et al. [13]	2019	Segmentation	Stroke	lesion
Vass et al. [14]	2022	Identification	Stroke	cranial cavity
Wu et al. [15]	2020	Segmentation	Stroke	lesion
Xue et al. [16]	2020	Segmentation	Stroke	lesion
Zavaliangos-Petropulu et al. [17]	2022	Identification	Stroke	hippocampus
Duan et al. [18]	2022	Sequence improvement	Speed	Speed
Iglesias et al. [19]	2023	Sequence improvement	Resolution	Resolution
Nishi et al. [20]	2020	Outcome prediction	Stroke	rankin scale
Karakis et al. [21]	2023	Outcome prediction	Stroke	upper limb motor function
Karthik et al. [22]	2020	Segmentation	Stroke	review
Fu et al. [23]	2019	Identification	Stroke	silent (from NLP)
Dyrba et al. [24]	2021	Identification	Alzheimer's	Alzheimer's

Supplementary Table 2: Paired two-sample t-tests comparing feature saliency in severe and nonsevere patients within different regions of interest (ROIs)

Grad-CAM++					SHAP			
ROIs	T	p	D	df	T	p	D	df
Lesion	-9.40	3.71E-17	-1.47	169	54.58	5.92E-108	8.9	166
Perilesional	-9.45	2.77E-17	-1.48	169	-20.8	4.33E-48	-3.38	166
Extralesional	-13.35	2.55E-28	-2.08	169	-47.68	6.22E-100	-7.71	166
LesionHomologue	11.84	6.15E-24	1.86	169	5.38	2.50E-07	0.88	166
PerilesionalHomologue	11.86	5.39E-24	1.86	169	5.36	2.76E-07	0.87	166
ExtralesionalHomologue	6.78	1.83E-10	1.06	169	4.44	1.59E-05	0.72	166

Supplementary Table 3: Lesion size and accuracy across severe patient subcategories

Subgroup	Lesion size	Accuracy	N
1	387.16	0.79	19
2	489	0.63	8
3	419.79	0.79	19
4	400.72	0.83	18
5	435.16	0.79	19
6	480.01	0.65	17
7	387.6	0.67	15

Supplementary Table 4: Lesion size and accuracy across nonsevere patient subcategories

Subgroup	Lesion size	Accuracy	N
1	118.4	0.65	20
2	118.6	0.7	20
3	141.32	0.82	22
4	138.05	0.74	19
5	99.74	0.79	19
6	128	0.81	16

Supplementary References

1. Şenbabaoğlu, Y., Michailidis, G., & Li, J. Z. (2014). Critical limitations of consensus clustering in class discovery. Scientific reports, 4(1), 6207.
2. John, C. R., Watson, D., Russ, D., Goldmann, K., Ehrenstein, M., Pitzalis, C., ... & Barnes, M. (2020). M3C: Monte Carlo reference-based consensus clustering. Scientific reports, 10(1), 1-14.
3. Hartigan, J. A., & Hartigan, P. M. (1985). The dip test of unimodality. The annals of Statistics, 70-84.
4. Dueck, D., & Frey, B. J. (2007, October). Non-metric affinity propagation for unsupervised image categorization. In 2007 IEEE 11th International Conference on Computer Vision (pp. 1-8). IEEE.Cao,
5. Y., & Wang, L. (2017). Automatic selection of t-SNE perplexity. arXiv preprint arXiv:1708.03229.
6. Mojiri Forooshani, P., Biparva, M., Ntiri, E. E., Ramirez, J., Boone, L., Holmes, M. F., ... & Goubran, M. (2022). Deep Bayesian networks for uncertainty estimation and adversarial resistance of white matter hyperintensity segmentation (Vol. 43, No. 7, pp. 2089-2108). Hoboken, USA: John Wiley & Sons, Inc..
7. Goubran, M., Ntiri, E. E., Akhavein, H., Holmes, M., Nestor, S., Ramirez, J., ... & Black, S. E. (2020). Hippocampal segmentation for brains with extensive atrophy using three-dimensional convolutional neural networks (Vol. 41, No. 2, pp. 291-308). Hoboken, USA: John Wiley & Sons, Inc..
8. Heit, J. J., Coelho, H., Lima, F. O., Granja, M., Aghaebrahim, A., Hanel, R., ... & Mont'Alverne, F. (2021). Automated cerebral hemorrhage detection using RAPID. American Journal of Neuroradiology, 42(2), 273-278.
9. Hilbert, A., Madai, V. I., Akay, E. M., Aydin, O. U., Behland, J., Sobesky, J., ... & Livne, M. (2020). BRAVE-NET: fully automated arterial brain vessel segmentation in patients with cerebrovascular disease. Frontiers in artificial intelligence, 3, 552258.
10. Li, T., Zou, Y., Bai, P., Li, S., Wang, H., Chen, X., ... & Zhou, G. (2021). Detecting cerebral microbleeds via deep learning with features enhancement by reusing ground truth. Computer Methods and Programs in Biomedicine, 204, 106051.
11. Meier, R., Lux, P., Jung, S., Fischer, U., Gralla, J., Reyes, M., ... & Kaesmacher, J. (2019). Neural network–derived perfusion maps for the assessment of lesions in patients with acute ischemic stroke. *Radiology: artificial intelligence*, *1*(5), e190019.
12. Ntiri, E. E., Holmes, M. F., Forooshani, P. M., Ramirez, J., Gao, F., Ozzoude, M., ... & Goubran, M. (2021). Improved segmentation of the intracranial and ventricular volumes in populations with cerebrovascular lesions and atrophy using 3D CNNs. Neuroinformatics, 1-22.
13. Sheth, S. A., Lopez-Rivera, V., Barman, A., Grotta, J. C., Yoo, A. J., Lee, S., ... & Giancardo, L. (2019). Machine learning–enabled automated determination of acute ischemic core from computed tomography angiography. Stroke, 50(11), 3093-3100.
14. Vass, L., Moore, M. J., Hanayik, T., Mair, G., Pendlebury, S. T., Demeyere, N., & Jenkinson, M. (2021). A Comparison of Cranial Cavity Extraction Tools for Non-contrast Enhanced CT Scans in Acute Stroke Patients. Neuroinformatics, 1-12.
15. Wu, W., Lu, Y., Mane, R., & Guan, C. (2020, July). Deep learning for neuroimaging segmentation with a novel data augmentation strategy. In 2020 42nd annual international conference of the IEEE engineering in medicine & biology society (EMBC) (pp. 1516-1519). IEEE.
16. Xue, Y., Farhat, F. G., Boukrina, O., Barrett, A. M., Binder, J. R., Roshan, U. W., & Graves, W. W. (2020). A multi-path 2.5 dimensional convolutional neural network system for segmenting stroke lesions in brain MRI images. NeuroImage: Clinical, 25, 102118
17. Zavaliangos-Petropulu, A., Tubi, M. A., Haddad, E., Zhu, A., Braskie, M. N., Jahanshad, N., ... & Liew, S. L. (2022). Testing a convolutional neural network-based hippocampal segmentation method in a stroke population. Human Brain Mapping, 43(1), 234-243.
18. Duan, C., Xiong, Y., Cheng, K., Xiao, S., Lyu, J., Wang, C., ... & Lou, X. (2022). Accelerating susceptibility-weighted imaging with deep learning by complex-valued convolutional neural

network (ComplexNet): validation in clinical brain imaging. European Radiology, 32(8), 5679-5687.

19. Iglesias, J. E., Schleicher, R., Laguna, S., Billot, B., Schaefer, P., McKaig, B., ... & Kimberly, W. T. (2022). Quantitative brain morphometry of portable low-field-strength MRI using super-resolution machine learning. Radiology, 220522.
20. Nishi, H., Oishi, N., Ishii, A., Ono, I., Ogura, T., Sunohara, T., ... & Miyamoto, S. (2020). Deep learning–derived high-level neuroimaging features predict clinical outcomes for large vessel occlusion. Stroke, 51(5), 1484-1492.
21. Karakis, R., Gurkahraman, K., Mitsis, G. D., & Boudrias, M. H. (2023). Deep learning prediction of motor performance in stroke individuals using neuroimaging data. Journal of Biomedical Informatics, 141, 104357.
22. Karthik, R., Menaka, R., Johnson, A., & Anand, S. (2020). Neuroimaging and deep learning for brain stroke detection-A review of recent advancements and future prospects. Computer Methods and Programs in Biomedicine, 197, 105728.
23. Fu, S., Leung, L. Y., Wang, Y., Raulli, A. O., Kallmes, D. F., Kinsman, K. A., ... & Liu, H. (2019). Natural language processing for the identification of silent brain infarcts from neuroimaging reports. JMIR medical informatics, 7(2), e12109.
24. Dyrba, M., Hanzig, M., Altenstein, S., Bader, S., Ballarini, T., Brosseron, F., ... & Teipel, S. J. (2021). Improving 3D convolutional neural network comprehensibility via interactive visualization of relevance maps: evaluation in Alzheimer’s disease. *Alzheimer's research & therapy*, *13*(1), 1-18.
