# Supplementary material for: Distinct brain morphometry patterns revealed by deep learning improve prediction of post-stroke aphasia severity
Source: Commun Med (Lond). 2024 Jun 12;4:115. doi: 10.1038/s43856-024-00541-8 (PMC11169346; doi:10.1038/s43856-024-00541-8)
Supplement: Supplementary file 3 — Description of Additional Supplementary Files [file 43856_2024_541_MOESM3_ESM.pdf]

Description of Additional Supplementary Files

File name- Supplementary Data set 1

File description- F1, precision, recall, and class accuracies for CNN over repeats of cross-validation

File name- Supplementary Data set 2

File description- Full distribution of permuted CNN model F1 scores

File name- Supplementary Data set 3

File description- t-SNE embeddings for each patient are presented along with median CNN predictions over repeats of cross-validation, true labels, and lesion categories for each patient

File name- Supplementary Data set 4

File description-F1, precision, recall, and class accuracies in each repeat of cross-validation for SVM models, including SVM without dimensionality reduction, SVM with PCA, and SVM with ICA

File name- Supplementary Data set 5

File description- F1 scores obtained from each iteration of cross-validation derived by applying weighted averages to the prediction probabilities from CNN and SVM models, across various weight combinations

File name- Supplementary Data set 6

File description- F1 scores obtained from each iteration of cross-validation, derived by stacking CNN and SVM predictions using an independent LDA model, across various hyperparameter (i.e., regularization) settings

File name- Supplementary Data set 7

File description- F1, precision, recall, and class accuracies in each repeat of cross-validation for the bestperforming stacked model (model with highest average F1 over repeats of cross-validation)

File name- Supplementary Data set 8

File description- F1, precision, recall, and class accuracies in each repeat of cross-validation for the SVM models trained on CNN Grad-CAM++ and deep SHAP maps

File name- Supplementary Data set 9

File description- Mean feature saliency values inside of 6 regions of interest for each participant correctly classified by the mean performing CNN (i.e., saliency based on Grad-CAM++) model based on the F1 score, and the corresponding SVM model (i.e., saliency based on SHAP); note, there are 5 patients for whom some regions of interest do not apply (i.e., patients with lesions so small that during the process of downsampling the data there are no 8mm voxels whose volume is more represented by lesioned native voxels than nonlesioned native voxels)

File name- Supplementary Data set 10

File description- Spatial Pearson correlation coefficients between Neurosynth topic meta-analysis maps and GradCAM++ maps generated for exemplars of patient clusters that each represent patients with similar Grad-CAM++ maps.

File name- Supplementary Data set 11

File description- Topic-based decodings presented in Figures 9 and 10 are available here.
