# Supplementary material for: Distinct brain morphometry patterns revealed by deep learning improve prediction of post-stroke aphasia severity
Source: Commun Med (Lond). 2024 Jun 12;4:115. doi: 10.1038/s43856-024-00541-8 (PMC11169346; doi:10.1038/s43856-024-00541-8)
Supplement: Supplementary file 15 — Reporting Summary [file 43856_2024_541_MOESM15_ESM.pdf]

Corresponding author(s): Alex Teghipco

Last updated by author(s): Mar 25, 2024

Reporting Summary

Nature Portfolio wishes to improve the reproducibility of the work that we publish. This form provides structure for consistency and transparency in reporting. For further information on Nature Portfolio policies, see our Editorial Policies and the Editorial Policy Checklist.

Statistics

For all statistical analyses, confirm that the following items are present in the figure legend, table legend, main text, or Methods section.

n/a	Confirmed	
☐	☒	The exact sample size (*n*) for each experimental group/condition, given as a discrete number and unit of measurement
☐	☒	A statement on whether measurements were taken from distinct samples or whether the same sample was measured repeatedly
☐	☒	The statistical test(s) used AND whether they are one- or two-sided<br>*Only common tests should be described solely by name; describe more complex techniques in the Methods section.*
☐	☒	A description of all covariates tested
☐	☒	A description of any assumptions or corrections, such as tests of normality and adjustment for multiple comparisons
☐	☒	A full description of the statistical parameters including central tendency (e.g. means) or other basic estimates (e.g. regression coefficient) AND variation (e.g. standard deviation) or associated estimates of uncertainty (e.g. confidence intervals)
☐	☒	For null hypothesis testing, the test statistic (e.g. *F*, *t*, *r*) with confidence intervals, effect sizes, degrees of freedom and *P* value noted<br>*Give P values as exact values whenever suitable.*
☒	☐	For Bayesian analysis, information on the choice of priors and Markov chain Monte Carlo settings
☒	☐	For hierarchical and complex designs, identification of the appropriate level for tests and full reporting of outcomes
☐	☒	Estimates of effect sizes (e.g. Cohen's *d*, Pearson's *r*), indicating how they were calculated

Our web collection on statistics for biologists contains articles on many of the points above.

Software and code

Policy information about availability of computer code

Data collection	No code was used for data collection.
Data analysis	The core models advanced in our work were generated using standard functions in PyTorch (version 2.0 with Python 3.10.11). The code we used to train, test, and interrogate the models is freely available at: https://github.com/alexteghipco/volDNN (DOI: 10.5281/zenodo.10849305). Code for consensus clustering was implemented in MATLAB (2022a) and can be accessed here: https://github.com/alexteghipco/consensusClustering (DOI: 10.5281/zenodo.10849272). Classical machine learning models were trained using standard functions in the statistics and machine learning toolbox in MATLAB (2022a). Code for a pipeline with nearly identical structure to what we employed (including for generating SHAP) can be found in another of our repositories: https://github.com/alexteghipco/StabilitySelection (see live code Tutorial3.mlx; DOI: 10.5281/zenodo.10231528). Any other code is available upon reasonable request to the corresponding author.

For manuscripts utilizing custom algorithms or software that are central to the research but not yet described in published literature, software must be made available to editors and reviewers. We strongly encourage code deposition in a community repository (e.g. GitHub). See the Nature Portfolio guidelines for submitting code & software for further information.

Data

Policy information about availability of data

All manuscripts must include a data availability statement. This statement should provide the following information, where applicable:

- Accession codes, unique identifiers, or web links for publicly available datasets
- A description of any restrictions on data availability
- For clinical datasets or third party data, please ensure that the statement adheres to our policy

Our data is available freely as part of our Aphasia Recovery Cohort (ARC) database: https://openneuro.org/datasets/ds004884/versions/1.0.1 (doi:10.18112/openneuro.ds004512.v2.0.0). A version of the dataset preprocessed in accordance with this manuscript has been made freely available: https://figshare.com/s/46012728175af8029c7b (10.6084/m9.figshare.23579943).
The minority of individuals used n our work that are currently unavailable in ARC (~20%) will be added to the growing database after it is ensured that the data is processed to comply with HIPAA regulation of Protected Health Information. Nifti brain images used in our figures are available on neurovault (https://identifiers.org/neurovault.collection:16012). Surface renderings from our figures can be downloaded from the above figshare link as a matlab file that can be loaded into our visualization software (see https://github.com/alexteghipco/brainSurfer; DOI: 10.5281/zenodo.7951955) to replicate the renderings exactly as we present them (i.e., all colormaps, thresholds, etc. used). See figure captions for source data for each figure. All other data is available from the corresponding author upon reasonable request.

Human research participants

Policy information about studies involving human research participants and Sex and Gender in Research.

Reporting on sex and gender	Participants were not recruited based on gender or sex, although we report general information about sex in our methods section (self-reported).
Population characteristics	*Describe the covariate-relevant population characteristics of the human research participants (e.g. age, genotypic information, past and current diagnosis and treatment categories). If you filled out the behavioural & social sciences study design questions and have nothing to add here, write "See above."*
Recruitment	*Describe how participants were recruited. Outline any potential self-selection bias or other biases that may be present and how these are likely to impact results.*
Ethics oversight	*Identify the organization(s) that approved the study protocol.*

Note that full information on the approval of the study protocol must also be provided in the manuscript.

Field-specific reporting

Please select the one below that is the best fit for your research. If you are not sure, read the appropriate sections before making your selection.

☐ Life sciences ☒ Behavioural & social sciences ☐ Ecological, evolutionary & environmental sciences

For a reference copy of the document with all sections, see nature.com/documents/nr-reporting-summary-flat.pdf

Behavioural & social sciences study design

All studies must disclose on these points even when the disclosure is negative.

Study description	This study analyzed quantitative retrospective chronic stroke data.
Research sample	Two-hundred and thirteen individuals (age = 57.98 +/- 11.34, 62% male) with chronic left strokes that participated in studies conducted at the Center for the Study of Aphasia Recovery (recruited from the community)
Sampling strategy	Convenience sampling, sample size determination does not apply as we analyzed retrospective cross-sectional data collected at a center across all studies that were conducted there.
Data collection	More detail is available in our methods. MRI data was collected in conjunction with behavioral data (The Western Aphasia Battery).
Timing	Data was collected between 2016 and 2022.
Data exclusions	All available data at the time of study analysis was used.
Non-participation	No participants dropped out or declined participation at this stage of the studies being analyzed (i.e.,intake sessions).

Randomization	Participants were not allocated into experimental groups for the data analyzed here.

Reporting for specific materials, systems and methods

We require information from authors about some types of materials, experimental systems and methods used in many studies. Here, indicate whether each material, system or method listed is relevant to your study. If you are not sure if a list item applies to your research, read the appropriate section before selecting a response.

Materials & experimental systems

n/a	Involved in the study
☒	☐ Antibodies
☒	☐ Eukaryotic cell lines
☒	☐ Palaeontology and archaeology
☒	☐ Animals and other organisms
☒	☐ Clinical data
☒	☐ Dual use research of concern

Methods

n/a	Involved in the study
☒	☐ ChIP-seq
☒	☐ Flow cytometry
☐	☒ MRI-based neuroimaging

Magnetic resonance imaging

Experimental design

Design type	Does not apply as no functional or longitudinal data was analyzed, only structural scans.
Design specifications	Does not apply as no functional data or longitudinal data was analyzed.
Behavioral performance measures	No behavioral data was collected during MRI.

Acquisition

Imaging type(s)	Structural T1 and T2-weighted images were collected.
Field strength	3T
Sequence & imaging parameters	The T1-weighted MPRAGE sequence has the following parameters: matrix = 256 × 256 mm, repetition time = 2.25 s, echo time = 4.11 ms, inversion time = 925 ms, flip angle = 9°, 1 x 1 x 1 mm, 192 slices, parallel imaging (GRAPPA = 2, 80 reference lines). The T2-weighted scan was acquired using sampling perfection with application-optimized contrasts and using different flip-angle evolution (SPACE) . Its parameters were: matrix = 256 × 256 mm, repletion time = 3200 ms, echo time = 567 ms, flip angle = variable, 1 x 1 x 1 mm, 176 slices, with parallel imaging (GRAPPA = 2, 80 reference lines).
Area of acquisition	Both scans were whole brain
Diffusion MRI	☐ Used ☒ Not used

Preprocessing

Preprocessing software	nii_preprocess and SPM were used for enantiomorphic healing, and FSL for normalization and tissue segmentation (i.e., FAST and fsl_anat pipelines in FSL v0.6.5.1)
Normalization	Data was normalized using the standard fsl_anat pipeline with standard settings after enantiomorphic healing (i.e. using both FNIRT and FLIRT). The only settings that were altered were the brain extraction settings, which were adjusted to get good brain extractions within individuals.
Normalization template	We used the MNI152 2mm template standard in FSL.
Noise and artifact removal	This does not apply.
Volume censoring	This does not apply.

Statistical modeling & inference

Model type and settings	This does not apply as we did not use a GLM. Instead, preprocessed tissue volumes output from FLS's FAST were used as inputs to train machine learning models to predict patients with severe aphasia (classification). Our core model was a single channel 3D convolutional neural network based on VGG architecture.

Effect(s) tested

This does not apply as we tested how well machine learning models could predict aphasia severity based on F1 scores computed over their predictions on out of sample data (within a repeated, nested, cross-validation scheme)

Specify type of analysis: ☐ Whole brain ☐ ROI-based ☒ Both

Anatomical location(s)

ROIs were based on the individual's specific lesion location, a perilesional area around the lesion, and the portion of the left (affected) hemisphere outside the lesion and perilesional area (i.e., the extralesional area). Right hemsiphere homologues for these regions were created by exploiting the symmetry of the MNI152 template (multiplying coordinates within the ROI by negative 1). The perilesional area was defined by dilating the lesion mask and subtracting the lesion mask from the resulting area.

Statistic type for inference
(See Eklund et al. 2016)

Uncorrected voxel-wise saliency maps for the models were investigated. Regional analyses limited the number of comparisons.

Correction

Uncorrected as saliency maps do not have clear corresponding p-values and regional analyses were limited in comparisons.

Models & analysis

n/a	Involved in the study
☒	☐ Functional and/or effective connectivity
☒	☐ Graph analysis
☐	☒ Multivariate modeling or predictive analysis

Multivariate modeling and predictive analysis

A VGG-style 3D CNN was used to predict aphasia severity from voxelwise morphometry data after downsampling the imaging data to 8mm. The model was tuned inside a nested cross-validation scheme where independent data was reserved for testing, training, and tuning (6-fold CV for testing and 8-fold CV for tuning models with stratification by lesion size quartiles). Evaluation was based on the F1 score to account for class imbalance.
